# AZT 5’-Phosphonates: Achievements and Trends in the Treatment and Prevention of HIV Infection

**Published:** 2013

**Authors:** А.L. Khandazhinskaya, E.A. Shirokova

**Affiliations:** Engelhardt Institute of Molecular Biology, Russian Academy of Sciences, Vavilov Str., 32, Moscow, Russia, 119991

**Keywords:** anti-HIV therapy, AZT 5'-phosphonates, depot forms, Nikavir, preclinical trials

## Abstract

Despite the numerous drawbacks, 3’-azido-3’-deoxythymidine (AZT, Zidovudine, Retrovir) remains one of the key drugs used in the treatment and prevention of HIV infection in both monotherapy and HAART. A strategy in searching for new effective and safe AZT agents among latent (depot) forms of AZT has yielded its first positive results. In particular, the sodium salt of AZT 5’-H-phosphonate (Nikavir, phosphazide) has demonstrated clinical advantages over parent AZT: first and foremost, lower toxicity and better tolerability. It can be effectively used for the prevention of vertical transmission from mothers to babies and as an alternative drug for HIV-infected patients with low tolerance to Zidovudine. Preclinical studies of another phosphonate, AZT 5’-aminocarbonylphosphonate, have demonstrated that it releases AZT when taken orally. Pharmacokinetic studies have shown a prolonged action potential. Based on the analysis of both toxicological and pharmacological data, AZT 5’-aminocarbonylphosphonate has been recommended for clinical trials.

## INTRODUCTION

In the last quarter of the 20^th^ century, AIDS morphed into one of the main threats to human health. The protracted efforts of researchers yielded a number of agents with anti-HIV activity which can be subdivided into several groups: 1) nucleoside and nonnucleoside reverse- transcriptase inhibitors of HIV (HIV RT ); 2) protease inhibitors; 3) integrase inhibitors; and 4) inhibitors of virus binding and penetration into cells. Nucleoside reverse transcriptase inhibitors (NRT I) are the most numerous group of anti-HIV agents used in clinical practice; the most commonly known members include 3’-azido-3’-deoxythymidine (AZT, Zidovudine, Retrovir), (-)-β-*L*-2’,3’-dideoxy-3’-thiacytidine (3TC , Lamivudine), (-)-β-*L*-2’,3’-dideoxy-3’-thia-5-fluorocytidine (L-FTC , Emricitabine), 2’,3’-dideoxyinosine (ddI, Didanosine), etc. [1]. The mechanism of action of these inhibitors includes intracellular triphosphorylation, followed by specific blocking of viral DNA synthesis catalyzed by HIV RT . However, all NRT Is have a number of serious drawbacks. Due to their pharmacokinetic properties and the low efficiency of intracellular conversions (e.g., as little as 0.3% of AZT is converted into the corresponding triphosphate in cells), high doses of the drug are needed, which in turn causes increasing toxicity. Furthermore, the high variability of the virus results in the rapid development of viral resistance [2, 3], which also narrows the range of therapy outcomes.

Among the clinical circumstances of AZT toxicity there are numerous hematological effects, suppression of bone marrow cell functions, liver disorders, myopathies, etc. [4, 5]. Mitochondrial toxicity of AZT causes hyperlactatemia and lipodystrophy in patients with AIDS [6, 7]. Since AZT is eliminated rather fast, this drug needs to be administered three times a day. After long-term therapy, AZT loses its efficiency with the development of viral resistance to it [8, 9]. Nevertheless, despite all the adverse effects, AZT remains the most widely used drug.

The currently used regimens of combined drug therapy (highly active antiretroviral therapy, HAART ) allow one to control HIV replication to a more significant extent, as compared to the drugs taken individually; however, they also require novel components that can be efficient and nontoxic.

One of the ways to enhance the efficacy of an antiviral drug is to synthesize its depot (latent) form; i.e., such a derivative that would release the active substance when undergoing chemical or enzymatic conversion in the organism [10]. Designing depot forms is a good means to reducing NRT I toxicity both by improving the pharmacokinetic parameters and by decreasing their affinity to mitochondrial transport proteins. This approach has been used in many laboratories in the search for novel anti-HIV agents. The following drugs illustrate the successful application of the depot forms of NRT I in practical medicine: Viread® (tenofovir disoproxil fumarate) and Nikavir® (sodium salt of AZT 5’-H-phosphonate, phosphazide) [11, 12].

## NIKAVIR (PHOSPHAZIDE), THE FIRST ACHIEVEMENT IN DESIGNING DEPOT FORMS OF AZTS

Nikavir was licensed in the Russian Federation in 1999 as an agent to be used for therapy in patients with AIDS and for the prevention of HIV infection [13-15]. Nikavir was designed as a result of lengthy studies devoted to the synthesis and investigation of antiviral agents headed by A.A. Kraevsky at the Engelhardt Institute of Molecular Biology, Russian Academy of Sciences [16, 17]:

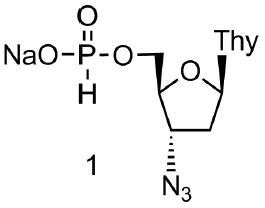



AZT 5’-H-phosphonate was synthesized in 1989 and tested on cell cultures infected with HIV-1 [16]; however, there was a significant discrepancy between the data obtained from the experiments conducted at different laboratories. It was initially reported that phosphazide** 1 **exhibited moderate anti-HIV activity and lower toxicity in a MT-4 cell culture as compared to AZT [18, 19]. Its selectivity index was higher than that of AZT. However, when phosphazide was later tested on three different cell lines (MT-4, CE M-SS and CE M-X 174), its activity was found to be lower than that of AZT by almost an order of magnitude [20]. It was reported in another paper [21] that the anti-HIV activity of compound **1 **in the cell cultures C8 and JM was 10–20 times higher vs. that of AZT. Contrariwise, according to [22], the selectivity indices of H-phosphonate **1 **were 1.5 and 15 times higher than those of AZT (IIIB and HXB2 strains of HIV in blood mononuclear cells were used). Despite these discrepant and disputable data, the research into phosphazide was continued, which made it possible to reveal its superiority over AZT in laboratory animal experiments [23].

Pharmacokinetic studies of phosphazide have shown the main difference from AZT: the pharmacokinetic profile of AZT after oral administration of phosphazide was considerably smoother as compared to the case when AZT was administered alone (*C*_max_ and *t*_max_ ~ 0.13 mg/l and 2–2.5 h, respectively, vs. 1.2 mg/l and 0.5–0.8 h for AZT). A lower peak concentration of AZT, which was observed after the administration of phosphazide, did not reduce antiviral efficacy but could facilitate a decrease in toxicity. This difference was used in clinical practice: a stable positive therapeutic effect (reduction of viral stress, immunorestoration, and a decrease in the risk of developing concomitant diseases) was observed. Phosphazide is well tolerated in both adults and children. No adverse effects that are typically observed in patients taking AZT (such as vomiting, nausea, headache, diarrhea, myalgia, anemia, thrombocytopenia, and neutrocytopenia) have been detected during phosphazide therapy [17].

A significant therapeutic efficacy and the safety of phosphazide were observed in HIV-infected patients receiving HAART . Various combinations of Nikavir with Didanozine and Nevirapine [24], with Didanozine and Ritonavir/Saquinavir [25], with Lamivudine and Efavirenz or a protease inhibitor (Atazanavir or Lopinavir/ Ritonavir) [26], etc. have shown good results. These HAART regimens have demonstrated higher efficacy in patients with concomitant diseases (anemias, chronic hepatitis B and C [27], hepatic cirrhosis, and tuberculosis [28]) as compared to regimens comprising Retrovir or Combivir. The essential advantage of Nikavir is that it is safe for patients with tuberculosis and liver pathologies of viral etiology, since most HIV-infected patients suffer from these opportunistic infections [27, 28].

Another clinical application of phosphazide is the chemoprophylaxis of mother-to-child transmission of HIV during pregnancy, peri- and postnatal periods. Phosphazide does not affect the course of the pregnancy in HIV-infected women, fetal maturity or viability. AZT from phosphazide is capable of efficiently penetrating the placenta; thus, equal AZT concentrations are maintained in the mother’s umbilical cord and blood. The use of Nikavir during a pregnancy (combined with Retrovir or Nevirapine during childbirth and the postnatal period) efficiently prevents vertical transmission of HIV [29, 30]. In some cases, Retrovir can be substituted by Nikavir because of a low hemoglobin level in the blood of a pregnant woman. The agent is well-tolerated; the hematologic indices have been restored in all such cases.

Thus, low toxicity and good tolerance of phosphazide make it a promising component for various examples of HAART . It can be efficiently used to prevent motherto- child transmission of HIV, to treat HIV infection (in particular, in patients with concomitant chronic viral hepatitis), and to prevent healthcare-worker infections. It is no coincidence that phosphazide has been recommended as a component of the preferred regimens of first-line antiretroviral therapy in the current edition of the Protocols on Treatment of and Care for People with HIV [31].

## 5’-AMINOCARBONYL PHOSPHONATES AS A TREND IN SEARCHING FOR NEW DEPOT FORMS OF AZT

Following the efforts focused on designing phosphonate depot forms of AZT, various types of compounds have been studied [32-37]. The class of AZT 5'-aminocarbonyl phosphonates substituted at the NH-fragment by various functional groups (C_6_H_13_, HOCH_2_CH_2_, H_2_NC H_2_CH_2_, Me_2_NC H_2_CH_2_, Me_3_N^+^CH_2_CH_2_, Me, Н, C_4_H_9_, PhCH_2_CH_2_) has turned out to have the highest potential [38-40].

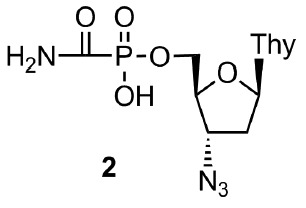



Antiviral experiments in HIV-infected MT-4 cells have demonstrated that AZT 5’-aminocarbonyl phosphonates inhibit viral replication with an efficiency an order of magnitude lower than that of AZT. Meanwhile, their toxicity (except for the methylamide derivative) is considerably lower than that of AZT. All the synthesized phosphonates have turned out to be stable in biological fluids (human blood serum, canine whole blood). The preliminary evaluation of the pharmacokinetic parameters after oral administration of AZT 5’-aminocarbonyl phosphonates in dogs has demonstrated that all the compounds can be metabolized to AZT. The shapes of the curves of concentration of the released AZT in blood plasma vs. time have been similar in all amides under study. The peak concentrations (*Cmax*) of AZT for phosphonates carrying C_6_H_13_, C_4_H_9_, PhCH_2_CH_2_ moieties at the NH-fragment, as well as that of nonsubstituted amide **2, **are 2.0, 0.8, 0.9, and 3.7 mg/l, respectively. Thus, AZT 5’-aminocarbonyl phosphate **2 **is the most efficient AZT donor within this group. This compound has been studied more thoroughly.

## AZT 5’-AMINOCARBONYL PHOSPHONATE

**Cell culture experiments**

The investigation of the antiviral activity of AZT 5’-aminocarbonyl phosphonate **2 **on a human lymphoblastoid cell line MT-4 has shown that its antiviral activity is inferior to that of AZT by about an order of magnitude and 3–4.5 times lower than that of phosphazide. However, its toxicity is considerably lower (by 34–50 and 12.5–15 times, respectively). Therefore, phosphonate** 2 **is characterized by a higher selectivity index than AZT and phosphazide [38-40].

The efficiency of penetration of phosphonate **2 **into cells is 10–100 times lower than that of AZT and approximately 6 times lower than that of phosphazide [23, 39]. This fact gives grounds to hypothesize that the decrease in anti-HIV activity and toxicity in a MT-4 cell culture as compared to the same indicators for AZT and phosphazide is associated with a decrease in the efficiency of its penetration into cells; i.e., there is a direct relationship between the penetration of phosphonate** 2**, phosphazide, and AZT into cultured cells and the antiviral properties of these compounds. It should be mentioned that both depot forms are appreciably stable in cell culture experiments, while being efficiently converted into AZT in the organism [39].

**Stability studies**

The stability of phosphonate **2 **in 100% human blood serum has turned out to be comparable to that of phosphazide: the half-life of both compounds is over 6 h [39]. Meanwhile, phosphonate **2 **is characterized by a considerably higher stability in canine whole blood at 37°С as compared to phosphazide (*Т*_1/2_ > 24 h vs. 3 h).

## PHARMACOKINETIC PARAMETERS AFTER SINGLE-DOSE ADMINISTRATION [41]

**Outbred dogs (average weight 22 ± 3.4 kg)**

It has been ascertained during a pharmacokinetic study of drug **2 **(capsules № 2; 250, 500 and 1000 mg or 10, 20 and 40 mg/kg) that most of it is metabolized into pharmacologically active AZT. *Table 1*lists the pharmacokinetic parameters of AZT released after a single-dose oral administration of phosphonate **2 **in dogs.

It was shown by comparing phosphonate **2 **with AZT and phosphazide (*Table 2*) that the peak AZT concentration in plasma during the administration of phosphonate** 2 **is lower, while the accumulation of AZT and the clearance time are longer. The pharmacokinetic parameters of AZT formed from phosphonate **2 **were close to those of phosphazide (*C*_max_ being 2.5 times lower; AUC being twice as low, but *t*_max_ and other parameters being higher). The maximum concentration of AZT after oral administration of phosphonate **2 **was attained after 4 h, which is twice as long as that after administration of AZT and 1 h longer than for phosphazide (*Table 2*).

**Table 1:** Pharmacokinetic parameters of 5’-aminocarbonyl phosphonate 2 and its major metabolite AZT after a singledose oral administration of capsules of 5’-aminocarbonyl phosphonate 2 to dogs at doses of 10, 20, and 40 mg/kg body weight

Dose of compound 2, mg	Tested compound	Pharmacokinetic parameters					
		C_max_, mg/l	t_max_, h	AUC , mg h/l	t_1/2_, h	MRT, h	Cmax/AUC, 1/h
10	2	0.31 ± 0.09	1.5 ± 0.25	0.47 ± 0.15	0.62 ± 0.1	2.43 ± 0.04	0.662 ± 0.066
	AZT	0.36 ± 0.24	4.7 ± 1.0	2.87 ± 1.56	4.57 ± 1.46	8.90 ± 5.34	0.119 ± 0.033
20	2	0.51 ± 0.18	1.6 ± 0.2	0.98 ± 0.44	0.81 ± 0.2	2.65 ± 0.16	0.561 ± 0.122
	AZT	0.69 ± 0.49	5.0 ± 1.7	6.0 ± 3.3	9.7 ± 4.3	12.0 ± 2.6	0.107 ± 0.023
40	2	0.51 ± 0.26	1.75 ± 0.27	1.25 ± 0.86	0.59 ± 0.2	2.87 ± 0.49	0.478 ± 0.117
	AZT	0.98 ± 0.56	6.0 ± 1.3	10.4 ± 6.1	7.0 ± 2.5	12.2 ± 1.4	0.100 ± 0.016

*Note. Here and in Tables 2, 3: AUCt – *area under the concentration-time curve; MRT – mean residence time; t_max_ – time needed to achieve the maximum concentration; C_max_ – maximum concentration of substance; t_1/2_ – half-life period.

**Table 2:** Comparison of the pharmacokinetic parameters of AZT after a single-dose oral administration of AZT 5’-aminocarbonyl phosphonate 2, phosphazide 1, or AZT to dogs at doses equivalent to 20 mg of AZT/kg body weight

Compound	C_max_, mg/l	t_max_, h	AUC , mg h/l	t_1/2_, h	MRT, h	CL, 1/h
2	0.74 ± 0.03	5	9.2 ± 0.2	9.6 ± 0.2	13.9 ± 0.2	27 ± 2.6
1	1.89±0.07	4	16.6 ± 0.3	7.2 ± 0.3	10.4 ± 0.5	15 ± 0.7
AZT	9.77 ± 0.3	2.5	58.8 ± 1.1	5.2 ± 0.5	7.5 ± 0.4	4.2 ± 0.3

It is noteworthy that the *t*_1/2_ and *t*_max_ AZT values in dogs increases in the following order: AZT < phosphazide < AZT 5’-aminocarbonyl phosphonate, which gives grounds for regarding AZT 5’-aminocarbonyl phosphonate** 2 **as a depot form of AZT with a long-term effect.

No AZT has been detected in the blood plasma of dogs after intravenous administration of phosphonate** 2 **at a dose of 50 mg (2–5 mg/kg body weight). The pharmacokinetic parameters of **2 **were as follows: AUC *_t_* = 2.19 mg·h/l, *t*_1/2_ = 0.35 h, MRT = 0.74 h, CL = 16.8 l/h, *V_ss_*= 12.4 l.

The bioavailability of 5’-aminocarbonyl phosphonate** 2 **administered orally at specified doses was 4.7%, while the bioavailability of AZT after **2 **had been administered orally was 8%, which is twice as low as that of phosphazide. The bioavailability of AZT during oral administration of this drug was six-fold higher than that in the case of phosphonate **2**. However, the high AUC value during the administration of AZT is associated with the excessive peak concentration in plasma, which decreases at a very fast rate. This causes toxicity and rapid emergence of drug-resistant viral strains. In turn, when administering phosphonate **2**, the difference between the maximum and minimum blood concentrations of AZT is significantly less pronounced, and this may reduce toxicity and inhibit the emergence of resistance.

**Chinchilla rabbits (average weight 3 ± 0.4 kg) [41]**

Studies of the pharmacokinetics of phosphonate **2 **(its aqueous solution was intragastrically administrated to rabbits) have also supported the assumption that it is a depot form of AZT. AZT has not been detected in the peripheral blood of rabbits (as well as dogs) that had received phosphonate **2 **intravenously. The original phosphonate** 2 **was the only product detected [17]. This fact confirms the hypothesis that AZT is formed as a result of the absorption of the original compound [17].

A comparison of the pharmacokinetic parameters of AZT and phosphonate **2 **after a single-dose oral introduction of phosphonate **2 **in rabbits at doses of 7, 70, and 200 mg/kg of body weight has demonstrated that AZT is present in the blood in all the cases. The shape of the concentration–time curves and the ratio between AZT and the original **2 **remains virtually unchanged as the dose is altered [39].

The results of a comparison of the pharmacokinetic properties of AZT after oral administration of single doses of AZT, phosphazide **1 **or phosphonate **2 **to rabbits are listed in *Table 3*. It should be mentioned that the shape of the dependence on the concentration of AZT released from phosphonate **2 **was considerably smoother; the *С*_max_ values of AZT released from phosphazide **1 **or phosphonate **2 **differs only twofold, while the AUC values of both phosphonates are rather close.

**Table 3:** Comparison of the pharmacokinetic parameters of AZT after a single-dose oral administration of AZT 5’-aminocarbonyl phosphonate 2, phosphazide 1, or AZT in rabbits at doses equivalent to 200 mg of AZT/kg body weight

Compound	C_max_, mg/l	t_max_, h	AUC, mg h/l	t_1/2_, h	MRT, h	CL, 1/h
2	3.75 ± 0.01	3.5	25.12 ± 1.08	3.66 ± 0.74	4.72 ± 0.08	44.22 ± 1.95
1	7.38 ± 3.08	2.0	22.99 ± 10.17	1.42 ± 0.12	3.02 ± 0.12	54.95 ± 22.85
AZT	39.64 ± 4.24	1.0	88.5 ± 25.5	2.13 ± 0.71	2.10 ± 0.30	9.40 ± 2.70

**Wistar rats and BALB/c mice**

No original phosphonate **2 **was detected in the blood plasma after it was taken orally by rats (body weight 200 ± 7 g) at a dose of 20 mg/kg. Only its metabolite AZT (characterized by the following pharmacokinetic parameters: AUC 0-t = 2.27 mg·h/l, MRT = 6.54 h, *t*_max_ = 4 h, *С*_max_ = 0.4 mg/l, *t*_1/2_ = 2.45 h and *С*_max_/AUC *_t_* = 0.176 h^-1^) was detectable.

Contrariwise, when phosphonate **2 **at a dose of 20 mg/kg was introduced intraperitoneally into rats (body weight 250 ± 10 g), phosphonate **2 **was the main compound detected, with trace amounts of its metabolite AZT. The pharmacokinetic parameters of phosphonate **2 **were as follows: AUC_0–*t*_ = 8.02 mg·h/l, MRT = 0.82 h, СL = 0.45 l/h, *t*_1/2_ = 0.42 h, and* V*_ss_ = 0.37 l. It is noteworthy that not only the original phosphonate **2**, but also 3.5% AZT were detected in the blood after phosphonate **2 **was administered intraperitoneally to mice at a dose of 6 g/kg of body weight.

Thus, AZT 5’-aminocarbonyl phosphonate **2 **releases AZT after being administered via different routes (orally, intragastrically or intraperitoneally) to experimental animals (mice, rats, rabbits and dogs) over a wide range of doses (7–6000 mg/kg of body weight) [17]. The pharmacokinetic parameters of phosphonate **2** and the AZT released from it in the blood plasma differ for different animal species. These differences can be attributed to the metabolic features of different animals and/or the route of drug administration.

The linear dependence of the pharmacokinetics of phosphonate **2 **with respect to its major metabolite AZT allows one to extrapolate the animal dose to a human dose. Thus, it can be expected for a single-dose oral administration of 600 mg of phosphonate **2 **that the AZT concentration in human blood plasma will be 100–115 ng/ml, with an appreciably gentle slope of the pharmacokinetic curve, which is considerably higher than the minimum AZT concentration achieved during regular (200 mg three times a day) oral administration of Zidovudine [42].

## PHARMACOKINETIC PARAMETERS DURING MULTIPLE-DOSE ADMINISTRATION OF AZT 5’-AMINOCARBONYL PHOSPHONATE [41]

The results of multiple-dose administration of AZT 5’-aminocarbonyl phosphonate show great promise as well.

Experiments on rabbits have shown a gradual accumulation of phosphonate **2 **in blood after a course of oral administration (solution – 1 g in 4–5 ml of water; administered after 6 and 18 h during 5 days) (*Fig.*). Furthermore, after the last dose had been administered (96 h after the onset of the experiment), AZT could be detected in human blood for 66 h (up to 162 h after the onset of the experiment).

Dogs (average weight 10.2 ± 1 kg) received compound** 2 **orally (600 mg on an empty stomach for 7 days, 24 h intervals). AZT was detected in plasma during the entire interval between the administrations of phosphonate** 2**. The identical *С*_0_ (0.17 ± 0.07 mg/l) and *С*_min_ (0.17 ± 0.07 mg/l) values on day 7 of administration indicate that the steady state was attained. The quasistationary concentration was 0.96 mg/l. The plasma levels of AZT at the steady state (2.82 ± 0.26) were characterized by an admissible fluctuation.

On day 7 of oral administration of phosphonate **2** capsules, AZT accumulation in the dog's organism was observed, manifesting itself in an increase in the AUC value (by 1.3 times as compared to day 1 of administration) and *C*_min_ (by 1.7 times as compared to day 1), as well as in *t*_max_ (from 2.7 to 4 h) and in *С*_max_ (from 2.45 to 2.75 mg/l blood plasma).

**Fig.**
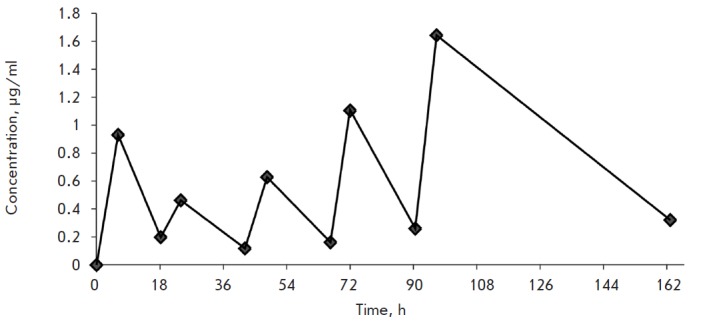
Concentration of AZT released in rabbit blood after multiple-dose administration of phosphonate** 2 **[17]

## DISTRIBUTION IN TISSUES [41]

The investigation of the tissue availability of novel drugs is an important stage in pharmacokinetic studies. Through distribution processes, a drug is delivered to its zone of action, where the drug interacts with structures that determine its effect. Measuring the tissue availability value allows one to quantitatively assess the intensity of the penetration of an active substance into peripheral tissues and the target organ.

The distribution of AZT, the metabolite of phosphonate** 2**, was studied in organs and tissues that differed in terms of blood supply, in the organs ensuring its elimination, and in the organ that is a potential action zone: strongly vascularized (liver, kidneys, spleen, lungs), moderately vascularized (skeletal muscles), and poorly vascularized (mesentery) tissues. AZT was detected in all these organs and tissues; its distribution over the organs was characterized by significant heterogeneity. The threshold of quantitative determination was 10 ng/ml. AZT could be detected in the blood plasma and organs of rats 12 h after a single-dose oral administration of phosphonate** 2 **at a dose of 100 mg/kg of body weight. Tissue availability of AZT in strongly vascularized organs (liver, spleen, lungs, and kidneys) was considerably higher than that in skeletal muscles and mesentery.

The AZT concentration generally decreased in a monophasic manner. The half-life period of the drug after oral administration was 3.9 h (blood plasma).

After a single-dose oral introduction of phosphonate** 2 **at a dose of 200 mg/kg, the original substance could not be detected in daily urine and feces, which can be attributed to intense biotransformation of the drug at the absorption stage. Only the major metabolite AZT was detected; it is eliminated as an insignificant percentage (4.11 and 0.04%, respectively) of the introduced dose of the drug.

## TOXICITY [41]

The results of toxicity studies conducted in mice have supported the assumption that slow accumulation in the blood and slower elimination of AZT released from phosphonate **2 **as compared to AZT administered directly and released after the introduction of phosphazide can decrease toxicity. Indeed, phosphonate **2 **is a low-toxicity compound, which has been confirmed by data obtained in experiments measuring acute toxicity (BALB/c mice and Wistar rats) and in chronic experiments (Wistar rats, breedless dogs). A single-dose administration of this substance to mice and rats at tested toxic doses (2000–50000 mg/kg) was accompanied by short-term agitation in animals replaced by distress, inertness and adynamia. The mean lethal dose (LD_50_) of phosphonate **2 **during intraperitoneal administration to mice was ≥ 5 g/kg, against 1.5 and 2.3 g/kg for AZT and phosphazide, respectively. LD_50_ for a single-dose intragastric administration of the substance to rats was higher than 40 g/kg.

The toxicity of phosphonate **2 **was studied under chronic experimental conditions in rats that had intragastrically received this substance at doses of 133 and 266 mg/kg daily for 3 months. The tested drug doses were 10- and 20-fold higher than the human daily dosage (13.3 mg/kg body weight). It turned out that compound** 2 **at tested doses was well-tolerated by animals and had no effect on the functional state of the main organs and systems of the organism (according to the results of biochemical tests) or hematological indicators. The absence of toxic lesions in internal organs and local irritating effects was confirmed in a pathomorphological study conducted after the conclusion of the experiment.

The toxicity of phosphonate **2 **in the form of capsules for oral administration, 200 mg, was assessed in dogs which intragastrically received the compound at a dose of 166 mg/kg (the 12.5-fold maximum recommended therapeutic dose for humans) daily for 4 weeks. It was found to be well-tolerated by the animals and to affect neither the functional state of the internal organs nor their hematological parameters (according to the data of biochemical tests). The absence of toxic lesions in internal organs and local irritating effects of phosphonate** 2 **in this pharmaceutical form after multipledose intragastric administration to dogs was confirmed through pathomorphological studies.

In order to assess the mutagenic properties of phosphonate** 2**, its ability to cause gene mutations in indicator strains of *Salmonella typhimurium *(Ames test), to cause chromosomal aberrations in the bone marrow cells of hybrid mice F1(CBAxC57Bl6), and to affect the number of dominant lethal mutations in mouse embryonic cells has been studied. It has been demonstrated that phosphonate **2 **at concentrations of up to 1 mg/dish does not increase the number of revertants in the Ames test in any statistically significant way.

When administered at doses 50-fold higher than the maximum recommended therapeutic dose for humans, phosphonate **2 **exhibited no mutagenicity in *in vivo* tests: it neither caused an increase in the number of chromosomal aberrations in mouse bone marrow cells nor affected the number of dominant lethal mutations in mouse embryonic cells.

When intragastrically administered at a dose of 133 mg/kg (10-fold maximum recommended therapeutic dose for humans) daily to Wistar rats (for 10 and 2 weeks to males and females, respectively), no effects of compound **2 **on the animal reproduction function was detected.

When intragastrically administered at a dose of 133 mg/kg daily to pregnant female rats on days 1–19 of gestation, phosphonate **2 **had no effects on the weight gain in pregnant rats, gestation duration, the number of corpus luteum, sites of embryo attachment, embryonic body weight, their craniocaudal dimension, indicators of pre- and post-implantation fetal death, or postnatal development of rat pups. Administration of compound **2 **caused neither malformations nor developmental disorders in embryos; in other words, it exhibits neither embryotoxic nor teratogenic effects.

The allergic properties of phosphonate **2 **have been studied in guinea pigs. Phosphonate **2 **was found to cause no anaphylactic shock when administered as a five-dose series at sensibilizing doses (133 and 266 mg/kg), followed by intragastric administration of an anaphylaxis-provoking dose (266 mg/kg) on days 14 and 21 after the sensibilization. At the doses and sensibilization schemes tested, the agent had no allergic effect in a type III hypersensitivity reaction on guinea pigs. Moreover, phosphonate **2 **was shown not to affect the popliteal lymph node reaction in mice.

When administered at doses of 166 and 332 mg/kg (12.5- and 25-fold maximum recommended therapeutic doses for humans), phosphonate **2 **affects neither the number of nuclear cells in spleen nor the hypersensitivity of the decelerated type reaction in mice. When administered at the highest of the doses tested (332 mg/kg), phosphonate **2 **reduced the primary immune response to a certain extent in F1(CBAxC57BI6) mice.

Thus, AZT 5’-aminocarbonyl phosphonate **2 **has been proved to be considerably less toxic as compared to the certified drugs Retrovir and Nikavir, to exhibit neither mutagenic nor allergic properties, to have no immunotoxicity, embryotoxicity or teratogenicity, and to have no effect on the reproductive functions of animals.

## CONCLUSIONS

The efforts of numerous researchers have resulted in the synthesis of over 100 novel potential depot forms based on a 5’-phosphonate modification of AZT; their anti-HIV activities have been tested. Nikavir® is the first AZT 5’-phosphonate that has been used as an anti-HIV drug. Preclinical studies of another phosphonate, AZT 5’-aminocarbonyl phosphonate, were completed recently, yielding rather encouraging results. Pharmacokinetic studies conducted on animals have demonstrated that when administered, phosphonate **2** is converted into AZT to a significant degree. The pharmacokinetic parameters of AZT attest to a long-term pharmacological effect.

An analysis of the combination of preclinical toxicological and pharmacological data gives grounds for recommending phosphonate **2 **for further clinical studies. The pharmacokinetic properties of this compound will presumably enable the administration of a drug based on it once a day, as opposed to Zidovudine, which is to be administered 2–3 times a day. Due to its lower toxicity, phosphonate **2 **can be used not only to prevent vertical HIV transmission, but also in children and HIVinfected patients with liver pathologies.

Thus, AZT 5’-aminocarbonyl phosphonate **2 **has an outstanding potential as an alternative to AZT and deserves further study.

## Abbreviations

HIV RT: human immunodeficiency virus reverse transcriptase
AZT: 3’-azido-3’-deoxythymidine
3ТС: (-)-β-L-2’,3’-dideoxy-3’-tiacytidine
L-FTC: (-)-β-L-2’,3’-dideoxy-3’-thia-5-fluoro-cytidine
НААRТ: high active antiretroviral therapy
LD50: mean lethal dose
AUC: area under the concentration-time curve
MRT: mean residence time
CL: total clearance
Vss: steady state volume of distribution
F: bioavailability
